# Evaluation of Entomopathogenic Fungi Against Chilli Thrips, *Scirtothrips dorsalis*

**DOI:** 10.1673/031.013.3101

**Published:** 2013-04-18

**Authors:** Steven Paul Arthurs, Luis Fernando Aristizábal, Pasco Bruce Avery

**Affiliations:** 1 Mid-Florida Research and Education Center, Department of Entomology and Nematology, University of Florida, IFAS, 2725 Binion Road, Apopka, FL 32703, USA; 2 Indian River Research and Education Center, University of Florida, Fort Pierce, Florida 34945, USA

**Keywords:** *Beauveria bassiana*, *Isaria fumosorosea*, *Metarhizium brunneum*, *Rosa* spp., sweet pepper

## Abstract

Commercial strains of entomopathogenic fungi were evaluated for control of chilli thrips, *Scirtothrips dorsalis* Hood (Thysanoptera: Thripidae), an invasive pest of ornamental and vegetable plants in the Caribbean and southeastern United States. In laboratory assays, LC_50_ values against adult *S. dorsalis* were 5.1 × 10^4^ CFU/mL for *Beauveria bassiana* GHA, with higher values 3.1 × 10^5^ for *Metarhizium brunneum* F52 and 3.8 × 10^5^ for *Isaria fumosorosea* Apopka 97. Second instars were comparatively less susceptible to all isolates, ostensibly due to moulting, with LC_50_ values of 1.1 × 10^8^, 7.0 × 10^5^, and 9.9 × 10^5^ CFU/spores per mL for GHA, F52, and Apopka 97 strains, respectively. In greenhouse cages, compared with controls, three applications of mycoinsecticides and other biorational insecticides at 7 to 14 day intervals reduced overall *S. dorsalis* populations on pepper plants *Capsicum annuum* cv. California Wonder: spinosad reduced populations by 94–99%, *M. brunneum* F52 by 84–93%, *B. bassiana* GHA by 81–94%, *I. fumosorosea* PFR-97 by 62–66%, and different horticultural oils by 58–85%. The proportion of marketable fruit was significantly increased by *M. brunneum* F52, *B. bassiana* GHA, and 2% SuffOil-X treatments. Slightly lower levels of control were observed in nursery tests with ornamental rose shrubs, *Rosa* sp. Red Double Knock Out®, during hot sunny conditions. Four applications reduced thrips populations over 10 weeks: spinosad by an average of 91%, *M. brunneum* F52 by an average of 81%, *B. bassiana* GHA by an average of 62%, SuffOil-X by an average of 50%, and *I. fumosorosea* PFR-97 by an average of 44%. The data show that mycoinsecticides can be used in management strategies for low to moderate populations of *S.* *dorsalis* and provide resistance management tools for the limited number of insecticides that are effective against this pest.

## Introduction

Chilli thrips, *Scirtothrips dorsalis* Hood (Thysanoptera: Thripidae), a recent invader in the Caribbean and southeastern United States, has emerged as a significant pest of landscape ornamental plants and poses a risk to several economically important agricultural crops (Seal et al. 2006b, 2010; Arthurs et al. 2009). Unlike many pest thrips, *S. dorsalis* attacks foliage, with both adults and larvae preferentially feeding on young leaves, buds, and fruits. Feeding causes distortion and discoloration, and severe infestations can lead to defoliation and stunted growth (Venette and Davis 2004). The broad host range and invasiveness of this species is a concern for regulatory agencies involved with USA agriculture and trade. Economically important hosts of *S. dorsalis* include banana, bean, cashew, castor, corn, citrus, cocoa, cotton, eggplant, grape, kiwi, litchi, longan, mango, melon, onion, passion fruit, peach, peanut, pepper, poplar, rose, sacara, soybean, strawberry, sweet potato, tea, tobacco, tomato, and wild yams (Venette and Davis 2004). *S. dorsalis* has been reported as a vector of several plant diseases, including tomato spotted wilt virus on peanut (Amin et al. 1981), peanut chlorotic fan virus (Campbell et al. 2005), and tobacco streak virus (Rao et al. 2003).

In 2004, the Florida Cooperative Agricultural Pest Survey program began a survey for *S. dorsalis* (Silagyi and Dixon 2006). Following its detection in Palm Beach County, Florida, in October 2005, this thrips species has rapidly spread throughout the state (Edwards et al. 2010). Shipments of infested ornamental plants from Florida are suspected to be responsible for recent populations of *S.* *dorsalis* reported in Texas (Ludwig and Bográn 2007), Louisiana (Ring 2012), and Georgia (Diffie and Srinivasan 2010). Nietschke et al. (2008) predicted the potential geographic distribution of *S. dorsalis* in North America would eventually extend north along the western coastal states to the Canadian border, as well as the entire Caribbean region.

A number of studies have evaluated chemical controls for *S. dorsalis.* Seal et al. (2006a) proposed a tentative management program for pepper production, including the application of chlorfenapyr at the beginning of infestation, followed by spinosad or imidacloprid application in four to seven days, and additional applications of these insecticides as needed at about seven-day intervals. In landscape tests with ornamental plants, Ludwig and Bográn (2007) reported that acephate, imidacloprid, or spinosad, provided control of *S. dorsalis.* However, in landscapes, exclusive reliance on chemical insecticides is probably not a sustainable option for *S. dorsalis* control, due to high costs, risks of pesticide resistance, and adverse effects on beneficial organisms and the environment (Reddy et al. 1992; Funderburk et al. 2000; Jensen 2000; Loughner et al. 2005). In Florida, all of these risk factors are enhanced by a long growing season, which allows multiple thrips generations each year.

Several studies have evaluated entomopathogenic fungi for use against western flower thrips, *Frankliniella occidentalis* Pergande, and other pest thrips (Vestergaard et al. 1995; Jacobson et al. 2001; Sengonca et al. 2006; Thungrabeab et al. 2006; Ansari et al. 2007, 2008). However, there is little information on the effectiveness of entomopathogenic fungi against *S.* *dorsalis.* Currently, several mycoinsecticides are registered for thrips control in North America. In this study, the pathogenicity of several commercially produced fungal strains against *S. dorsalis* life stages were evaluated in the laboratory. Several reduced risk insecticides were compared against infestarions of *S. dorsalis* in greenhouse and nursery studies.

## Materials and Methods

### Source of insects and plants

*S. dorsalis* were obtained from wild populations on ornamental rose bushes in Seminole County, Florida, in 2008, and reared on cotton (Deltapine 493 Conventional) plants in an insectary room maintained at 25 ± 2° C, 70% RH, and under a 12:12 L:D photoperiod. Cotton and pepper plants used in tests were germinated from seed in trays in a pest free greenhouse and transplanted at the sixth to eighth true leaf stage into 15 cm diameter pots using Fafard Growing Mix 2 (Conrad Fafard, Inc., www.fafard.com). Cotton and pepper plants were fertilized weekly with liquid 12-4-8 NPK (Miracle-Gro, Scotts, www.scotts.com) Ornamental roses, *Rosa* sp. Red Double Knock Out®, were acquired from a local nursery and held for 12 months to eliminate insecticide residues.

### Experimental treatments

The following fungal preparations were used in tests: *Beauveria bassiana* (Balsamo-Crivelli) Vuillemin strain GHA containing 4.4×10^10^ conidia per g (BotaniGard® 22WP, BioWorks, www.bioworksinc.com), *Isaria fumosorosea* Wise (= *Paecilomyces fumosoroseus*) Apopka strain 97 containing 10^9^ CFU(blastospore)/g (PFR-97 20% WDG, Certis, certisusa.com), and an oil-based formulation of *Metarhizium brunneum* Petch strain F52 containing 5×10^9^ conidia/mL (Met52 EC, Novozymes, www.novozymes.com). Germination rates of tested materials were > 80% on PDA after 24 hours at 25° C.

### Laboratory bioassay

Fungal preparations were screened against *S. dorsalis* using a modification of the leaf-disc method of Ugine et al. (2005). All materials were suspended in distilled water with a magnetic stirrer, and the following concentrations were prepared using an improved Neubauer hemocytometer and serial (×10) dilution, 10^3^, 10^4^, 10^5^, 10^6^, 10^7^, 10^8^, and 10^9^ conidia/blastospores per mL. A spreading agent, Tween 80 at 0.05% v/v, was included in all cases, and samples sonicated for 2 minutes to break fungal chains. Fungal suspensions were applied using a Potter spray tower (Burkard Scientific, www.burkardscientific.co.uk) to the abaxial surface of leaf discs (2 cm diameter), which were removed with a sterilized cork borer from six-week-old cotton plants. Spray volumes were calibrated at 2.5 µl per cm^2^ on the leaf surface, i.e., delivering between 2.5 and 2.5 × 10^6^ conidia/blastopores per cm^2^ over the range of concentrations. The highest concentrations were filtered through cheesecloth; however, since the larger particulate size of the formulating material for *I. fumosorosea* (bran) sometimes clogged the spray tower nozzle, the highest concentration was dropped in that treatment. Leaf discs were air-dried and placed individually in 30 mL plastic cups (Solo, www.solocup.com) containing 4 mL of water agar covered with a single 3.5 cm diameter filter paper. This setup preserved high humidity to maintain leaf turgor while also reducing water droplets that could trap insects. Each cup was infested with 10 adults or 20 second instar thrips using an entomological paintbrush. Cups were capped with a tightly fitting lid and incubated at 25 ± 1° C, 80% RH, and with 16:8 L:D photoperiod. Thrips mortality was assessed under a dissecting microscope after seven days. The proportion of cadavers expressing symptomatic development sporulation was noted. There were five cups per fungus concentration/life stage tested, and the study was repeated on three occasions for each fungus species.

### Greenhouse cage study

All mycoinsecticides were tested within label rates against artificially established *S. dorsalis* populations on sweet pepper, *Capsicum annuum* cv. California Wonder, under greenhouse conditions. Prior to tests, sixweek-old plants (pest free) were infested with 15 adult thrips, followed by an additional 10 after one week (25 thrips/plant total), using the methods of Dogramaci et al. (2011). Since compact plants were easier to manipulate inside cages, uniconazole-P (Sumagic, plant growth regulator, Valent, www.valent.com) was applied at 2 µl/L to limit stem elongation. Plants were maintained inside cages (two plants per cage), consisting of a PVC frame (60 × 60 × 60 cm) covered with nylon mesh (0.36 mm hole size) and fitted with a sleeve for access.

In the first trial, conducted in fall 2010, *M. brunneum* F52 EC (2.1 ml/L applying 10^10^ spores/L), PFR-97 WDG (2.1 g/L applying 2.1 × 10^9^ spores/L) and BotaniGard WP (2.4 g/L applying 10^11^ spores/L), and a highly refined horticultural oil (SuffOil-X, BioWorks) at 2% v/v and spinosad (Conserve® SC, Dow AgroSciences, www.dowagro.com) at 0.47 ml/L were compared. There were six replicate cages per treatment (36 cages total), arranged randomly inside a greenhouse bay. Treatments were applied one week after the second thrips infestation, when eggs and F1 larvae were present. Foliage was sprayed to run-off, using a backpack sprayer (Flo-Master 1101HD, 3.8 L capacity) fitted with a cone nozzle. Concurrently, the soil surface was sprayed around the base of the plant to target any additional nonfeeding stages. An application rate equivalent to 1,000 L/hectare was applied, and a wetting agent (Tween 80 at 0.05% v/v) was added to all treatments. Controls were treated with water and Tween. Plants were isolated for spraying in order to manage drift to adjacent plants and cages. To improve environmental conditions for fungal germination, treatments were applied late in the day (after 18:00) when cooling fans had stopped. Cages were lightly irrigated using overhead misters immediately following spraying, which were noted to maintain > 95% RH in the canopy for approximately 12 hours, but did not result in surface leaf wetness. Two additional applications were made after 7 and 20 days.

Thrips were sampled from the three uppermost terminal leaves (Seal et al. 2006b) at five weekly intervals starting immediately before the first treatments were applied. Plants were scored according to the injury scale described by Kumar et al. (1996): (0) no symptoms; (1) terminal three to four leaves showing tiny eruptions in the inter-veinal area; (2) terminal three to four leaves showing upward curling along leaf margin; (3) severe scarring of terminal and a few basal leaves; (4) stunted plants, leaves severely curled and leaf area greatly reduced; (5) plants with no leaves and only stem remaining. At the end of each experiment, pepper fruits were rated for marketability based on visible insect damage (deformation). The trial was repeated in the spring, with the following modifications: only a single plant was used per cage (infested once with a lower rate of 15 thrips), a different horticultural oil (Year-Round™ Spray Oil, Summit Chemical, www.summitchemical.com) was used at 1% v/v, and evaluations were made over six weeks.

Shade temperature and relative humidity were monitored with a Hobo H8 Pro Series loggers (Onset, www.onsetcomp.com). Conditions inside greenhouse cages averaged 22.8° C (range 14.8–40.1) and 78.5% RH (range 15–100) over the tests.

### Nursery study

A simulated nursery study was conducted from late spring through summer in 2011 using ornamental roses, *Rosa* sp. ‘Red Double Knock Out®, transplanted into 20 L pots containing multipurpose compost and maintained on landscape fabric. Plants were fertilized every other month with 25 g of slow release granules (Scotts Rose & Bloom® 12-4-8) and watered daily (2 L/pot) during the hotter months through drip irrigation. Plants, pruned two weeks prior to encourage new growth, were infested with 30 adult thrips. Ten adults were attached to three separate terminals using previously described methods (Dogramaci et al. 2011). The same treatments used in the greenhouse study were evaluated, but this time set up as a randomized block design. There were six blocks, each one containing one plant for each treatment. Blocks were separated by 10 m, with yellow sticky cards between blocks to monitor thrips movement. Treatments were applied 15 days postinfestation, when *S. dorsalis* larvae were observed, with additional applications made after 8, 31, and 42 days. Foliage was sprayed to run-off, using a backpack sprayer with a cone nozzle, while an equivalent volume was applied to the soil surface to target nonfeeding stages (equivalent to 1000 L/hectare). A nonionic wetting agent (R-11, Wilbur-Ellis, www.wilburellis.com) was added to all treatments at 0.15% v/v. Controls were treated with water and wetting agent only. Treatments were applied late in the day (after 18:00), and plants were isolated to manage drift. Evaluations were made *in situ* weekly over 10 weeks, starting shortly prior to spraying. Thrips' life stages were counted with a 10× field magnification visor from three randomly selected terminals per plant. The proportion of new plant terminals with thrips damage was also assessed from 10 terminals per plant.

**Table 1.**
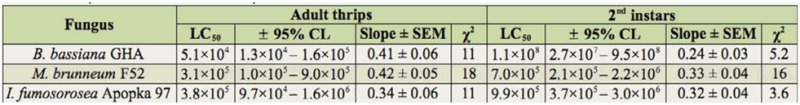
Estimates of the median lethal concentration (spores/mL) of three entomopathogenic fungi applied against different *Scirtothrips dorsalis* life stages in leaf disc assays. Data based on three replicate tests (50 adults or 100 larvae/test); all probit estimates were adjusted for control mortality.

### Data analysis

In laboratory bioassays, mortality estimates averaged for each test date (n = 3) were compared using univariate analysis of variance (ANOVA). Probit analysis of the log10 concentration was used to estimate LC_50_ along with fiducial limits and slope (SPSS for windows v. 17, IBM, www.ibm.com). In greenhouse and nursery tests, treatments were compared using a repeated measures ANOVA having six treatments and between 5 and 10 time intervals (SPSS). Means were separated using Tukey's HSD tests in the repeated measures model. All data were checked for normality and, if needed, normalized through log (n + 1) or arcsine (proportion data) prior to analysis.

## Results

### Laboratory assay

**Figure 1.**
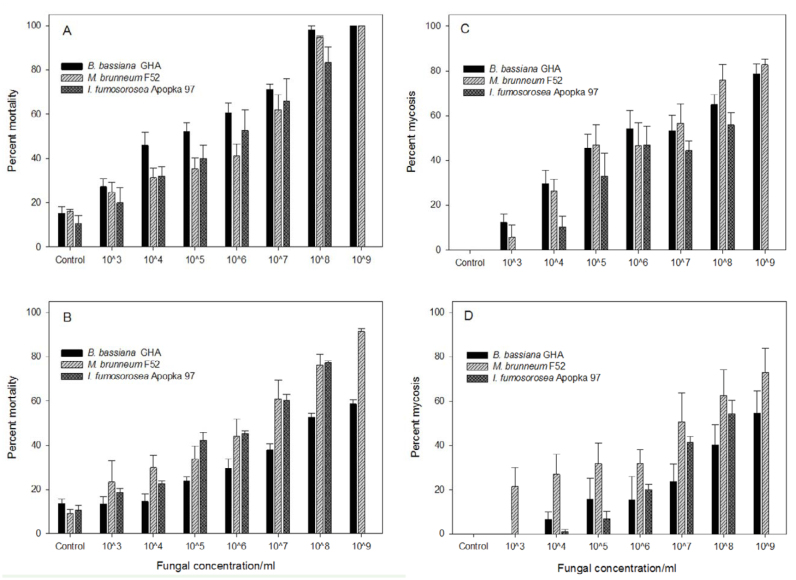
Response of *Scirtothrips dorsalis* to three fungal entomopathogens in leaf disc assays; (A) total mortality of thrips exposed as adults, (B) total mortality of thrips exposed as larvae, (C) proportional mycosis among adults, (D) proportional mycosis among larvae. Data show mean ± SEM of three tests (five replicates per test). High quality figures are available online.

All fungal strains caused significant thrips mortality with a clear dose-response (Figure 1A, B). Adult thrips were more susceptible than larvae (F_1, 132_ = 9.4, *p* < 0.005), with a further two-way interaction with fungal species and thrips life stage (F_2, 132_ = 3.2, *p* < 0.05). This interaction is explained by comparisons of the LC_50_, which shows a greater disparity in susceptibility between thrips adults and larvae for *B. bassiana* (Table 1). The proportion of dead thrips sporulating with symptomatic mycosis followed a doseresponse (Figure 1C, D). Overall, higher rates of sporulation were observed among thrips exposed as adults compared with larvae (F_1, 132_ = 8.3, *p* < 0.005), with differences according to fungal species (F_2,132_ = 5.7, *p* < 0.005), although the interaction with fungal species and life stage was not significant (F_2_, _132_ = 1.4, *p* = 0.24).

### Greenhouse cage study

**Figure 2.**
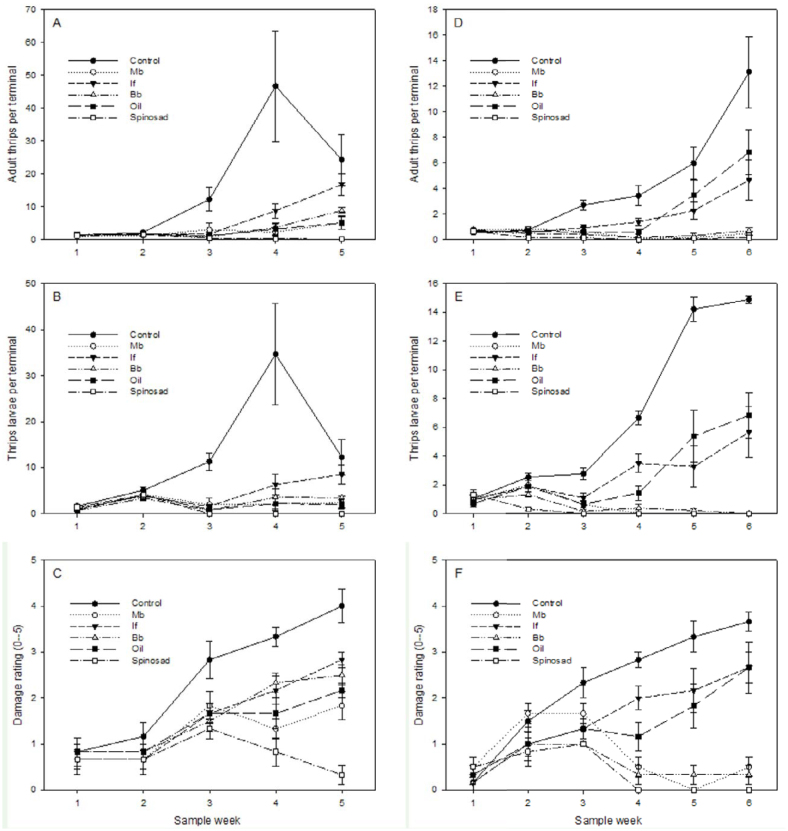
Thrips counts and plant damage in greenhouse tests with caged pepper plants; (A–C) adults, larvae, and plant damage in fall test, (D–F) adults, larvae, and plant damage in spring test. Data are mean ± SEM from plants in six replicate cages. High quality figures are available online.

**Figure 3.**
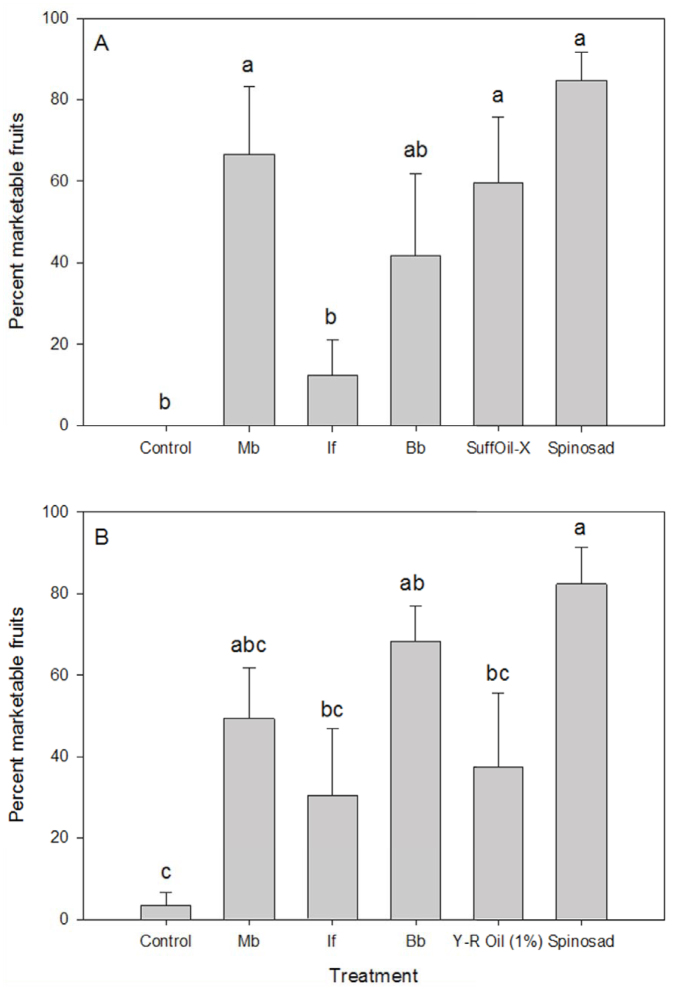
Final proportion of marketable pepper fruits (mean ± SEM) in greenhouse studies: (A) fall, (B) spring tests. High quality figures are available online.

In the fall, thrips developed through at least two complete generations, reaching ≥ 80 individuals per pepper terminal and causing extensive deformation and defoliation of control plants by the end of the study (Figure 2A–C). Insecticide treatment significantly affected both the number of adult thrips and thrips larvae sampled on plant terminals (F_5,30_ = 16.5, *p* < 0.0001, and F_5,30_ = 17.0, *p* < 0.0001, respectively, in repeated measures ANOVA). Compared with controls, thrips populations were significantly reduced (*p* < 0.05, Tukey HDS in the repeated measures model) by the following treatments: spinosad (average 94% reduction), followed by SuffOil-X (85%), *M.* *brunneum* (84%), *B. bassiana* (81%), and *I. fumosorosea* (66%). Intermediate expression of thrips feeding symptoms (rating 1.8–2.8) was recorded in the insecticide treatments, with the exception of spinosad, which remained low (< 0.3) (Figure 2C). Although the initial feeding damage likely impaired fruit set before treatments were first applied, increased levels of marketable fruit in several treatments, notably spinosad, *M. brunneum*, and SuffOil-X, were observed (Figure 3A).

Broadly similar findings occurred in the spring test, although thrips populations were somewhat lower compared with the fall, and reduced effectiveness was observed with the different horticultural oils (Figure 2D–F, 3B). Overall, insecticide treatments were highly significant (F_5,__30_ = 45.5, *p* < 0.0001), with thrips populations significantly reduced by the following: spinosad (average 99% reduction compared with control plants), *B. bassiana* (94%), *M. brunneum* (93%), *I. fumosorosea* (62%), and 1% oil (58%).

### Nursery study

**Figure 4.**
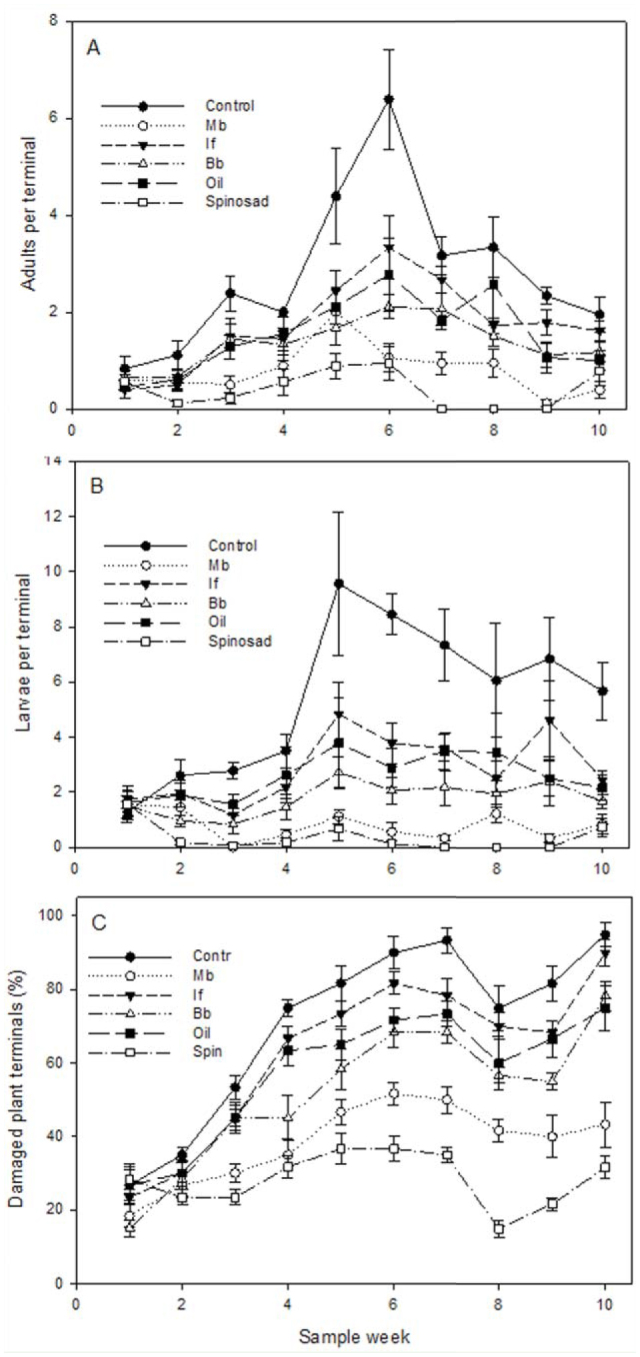
Weekly thrips counts, (A) adults and (B) larvae, and (C) damaged plant terminals from knockout roses treated with mycoinsecticides and other biorational materials in nursery study. Data are mean ± SEM of six replicate shrubs. High quality figures are available online.

Thrips developed through three to four generations, with populations peaking in weeks five and six on control plants and gradually declining over the remainder of the study (Figure 4). Repeated measures ANOVA revealed that treatment applied, including controls, significantly affected both the number of adult thrips (F_5, 30_ = 37.2, *p* < 0.0001) and thrips larvae (F_5,__30_ = 42.1, *p* < 0.0001) sampled on plant terminals. Compared with control plants, thrips (adults and larvae combined) were significantly reduced (*p* < 0.05, Tukey HDS in the repeated measures model) by the following treatments: spinosad (average 91% reduction with respect to control plants), *M. brunneum* (81%), *B. bassiana* (62%), SuffOil-X (50%), and *I*. *fumosorosea* (44%). Thrips damage, observed on > 95% of new rose terminals on untreated plants (Figure 3C), was also affected by treatments (F_5,__30_ = 51.7, *p* < 0.0001). Although posthoc tests revealed that less damage occurred on all treated plants compared with controls (*p* < 0.05 in the repeated measures model), a relatively high proportion of terminals were still affected, with only spinosad and *M. brunneum* having 50% of terminals damaged by the end of the study. The predator *Orius insidiosus* Say was observed throughout all plots during the study, although its impact was not quantified. Conditions remained generally hot and sunny (up to 1030 W/m^2^) with some afternoon showers; average hourly measurements ranged from 13.2 to 37.3° C, 28 to 96% RH, with a total of 30.4 cm rainfall during the study.

## Discussion

This study highlights the potential of mycoinsecticides for management of *S. dorsalis* in vegetables or ornamental plants. Both BotaniGard and PFR-97 are registered in North America for greenhouse and nursery use against thrips and other soft bodied pests, including aphids and whiteflies. *Metarhizium* strain F52 is approved for non-food use in greenhouses, nurseries, and limited outdoor sites. In particular, respectable rates of control on pepper (> 80% population reduction) were obtained from *B. bassiana* and *M. brunneum* applied within label rates in greenhouses. The increasing numbers of thrips in all treatments at the end of this study may have been exacerbated by adults migrating out of control cages after destruction of host plants, since thrips were intercepted on sticky cards hung between cages at this time (at < 2mm, adult *S. dorsalis* can fit though most insect screens). It would need to be confirmed whether the humidity inside greenhouse cages (elevated approximately 3% compared with outside) enhanced efficacy of fungal treatments compared with an open crop.

Slightly less effective control was achieved on roses maintained outside during summer conditions. Given that *S. dorsalis* primarily feeds on developing foliage and unopened flower buds, it is likely that higher UV levels were detrimental to the fungal persistence in these tests (Braga et al. 2001; Fernandes et al. 2007; Zimmermann 2008). However, the possibility of thrips movement between control and treated plants in the nursery study cannot be excluded; hence, better control might be expected under operational conditions where reinfestation from untreated plants is minimized.

Although application strategy was not quantified, the spraying of soil surfaces may be beneficial by targeting the developing prepupal and pupal stages. Application of entomopathogenic fungi to container plants though soil drenches or pre-mixing with potting compost is an effective strategy against *F. occidentalis* (Brownbridge 1995; Helyer et al. 1995; Ansari et al. 2008). The species/strain of fungus may influence the level of control achieved against thrips. In laboratory tests, Ansari et al. (2008) reported that *M. brunneum* strains V275 and ERL700 were the most effective, causing 85 to 96% mortality of *F. occidentalis* larvae and pupae 11 days after inoculation in the soil, compared with 51 to 84% mortality in four other *M. brunneum* strains, 54 to 84% mortality from two *B. bassiana* strains, 63 to 75% mortality from two *I. fumosorosea* strains, and 15 to 54% for the insecticide Fipronil.

There are few reports of entomopathogenic fungi being used against *S. dorsalis.* However, compared with our data, Seal and Kumar (2010) reported relatively less effective control of *S. dorsalis* treated with *B. bassiana* GHA. In insecticide screening tests with Jalapeño peppers under greenhouse conditions, label rates of BotaniGard applied to the foliage reduced *S. dorsalis* larvae by about 50% at 5 days after treatment, but not at 10 days after treatment (Seal and Kumar 2010). Differences in experimental procedures may explain these differences, since in the latter tests the soil surface was not apparently treated, and pepper plants were maintained adjacent to infested cotton plants, which may have encouraged more rapid reinfestation.

Fungal pathogens have been recovered from *S. dorsalis* in its native range. Two strains of *Fusarium* spp. and *Neozygites floridana* were isolated from cadavers of *S. dorsalis* in chilli pepper fields in India (Mikunthan and Manjunatha 2006). The authors cultured both *Fusarium* isolates on media and subsequently obtained LC_50_ value of 2.7 × 10^7^ and 7.6 × 10^7^ spores/mL for *Fusarium incarnatum* (formerly *semitectum*) (Desm.) Sacc and *Fusarium* sp. isolate GM 15, respectively, applied against second instar *S. dorsalis.* In further tests, the researchers applied oilformulation of the more virulent *F. incarnatum* strain against *S. dorsalis* in field-grown chilli peppers at 35, 50, and 70 days post-transplanting (Mikunthan and Manjunatha 2008). The authors attempted to improve the micro-environmental conditions for fungal infection and transmission through intercropping chilli peppers with taller companion plants. Although the lack of unsprayed controls prevented the impact of the fungus being addressed directly, the authors observed improvements in overall yield of chilli peppers in treated plots planted with chilli-sorghum, chilli-cotton-chilli, and chilli-red gram, and suggested that chilli-cotton-chilli cropping systems are favorable environmental conditions for managing *S. dorsalis* and broad mites using *F. incarnatum.*

Relatively more species and strains of entomopathogenic fungi have been tested against *F. occidentalis.* Brownbridge (1995) tested more than 150 isolates and concluded that *M. brunneum*, *B. bassiana*, and *Lecanicillium lecanii* R. Zare and W. Gams had the highest level of insecticidal activity. Sengonca et al. (2006) tested 41 isolates from 25 species and 11 genera of entomopathogenic fungi in Thailand against first instar *F. occidentalis* on bean leaves. Among the 14 most virulent isolates, LC_50_ values of *Beauveria* spp. ranged from 2.4 × 10^4^ to 5.9 × 10^6^ conidia/mL, *Metarhizium* spp. from 2.0 × 10^4^ to 5.0 × 10^5^ conidia/mL, and *Isaria* spp. from 3.9 × 10^4^ to 5.5 × 10^6^ conidia/mL. The latter two values reported by Sengonca et al. (2006) compare with our laboratory-based estimates for second instar *S. dorsalis* reared on cotton (i.e., within 95% CL from Table 1). However, higher LC_50_ values were observed among *S.* *dorsalis* larvae exposed to *B. bassiana* in the present study, possibly due to its shorter developmental period compared with *F. occidentalis.* The difference in host plant used in the present laboratory studies and those of Sengonca et al. (2006) may have influenced the results. Thungrabeab et al. (2006) reported that *F. occidentalis* reared on cotton were significantly less susceptible to infection by *B. bassiana* compared with those reared on bean plants, ostensibly due to sequestered gossypol and/or other allelochemicals in the cotton plants (Poprawski and Jones 2000).

The observation that *S. dorsalis* larvae were less susceptible to mycosis than adults is consistent with the inoculum being shed with the exuvium during ecdysis. Vestergaard et al. (1995) also reported lower susceptibility of *F. occidentalis* larvae compared with adults exposed to *M. brunneum* V275 (27% versus 100% mortality). Evidence that ecdysis may provide a resistance mechanism to fungal infection was provided by Ugine et al. (2005), who demonstrated that early second instar *F. occidentalis* became progressively less susceptible to *B. bassiana* as they aged within the instar. It is hypothesized that a slower germination of *B. bassiana* on the *S. dorsalis* larval cuticle may have reduced its efficacy compared with other fungal strains.

In conclusion, mycoinsecticides may be used to manage *S. dorsalis* and provide resistance management tools for spinosad or other insecticides. Mycoinsecticides may be most effective in pest managements programs integrating beneficial arthropods, or in greenhouse crops where favorable environmental conditions (high humidity and low UV exposure) can be manipulated (Jacobson et al. 2001; Down et al. 2009). Additional research to optimize entomopathogenic fungi, for example through formulation, is warranted. Other candidate entomopathogens include entomopathogenic nematodes (Jagdish and Purnima 2011), while cultural methods include reduced fertilizer inputs and removing pupations substrates (Duraimurugan and Jagadish 2004).
